# Structural and functional leaf diversity lead to variability in photosynthetic capacity across a range of *Juglans regia* genotypes

**DOI:** 10.1111/pce.14370

**Published:** 2022-06-20

**Authors:** Mina Momayyezi, Devin A. Rippner, Fiona V. Duong, Pranav V. Raja, Patrick J. Brown, Daniel A. Kluepfel, J. Mason Earles, Elisabeth J. Forrestel, Matthew E. Gilbert, Andrew J. McElrone

**Affiliations:** ^1^ Department of Viticulture and Enology University of California Davis California USA; ^2^ USDA‐ARS, Horticultural Crops Research Unit Prosser Washington USA; ^3^ Department of Plant Sciences University of California Davis California USA; ^4^ USDA‐ARS, Crops Pathology and Genetics Research Unit Davis California USA

**Keywords:** CO_2_ conductance, leaf anatomy, photosynthesis, walnut wild accessions

## Abstract

Similar to other cropping systems, few walnut cultivars are used as scion in commercial production. Germplasm collections can be used to diversify cultivar options and hold potential for improving crop productivity, disease resistance and stress tolerance. In this study, we explored the anatomical and biochemical bases of photosynthetic capacity and response to water stress in 11 *Juglans regia* accessions in the U.S. department of agriculture, agricultural research service (USDA‐ARS) National Clonal Germplasm. Net assimilation rate (*A*_n_) differed significantly among accessions and was greater in lower latitudes coincident with higher stomatal and mesophyll conductances, leaf thickness, mesophyll porosity, gas‐phase diffusion, leaf nitrogen and lower leaf mass and stomatal density. High CO_2_‐saturated assimilation rates led to increases in *A*_n_ under diffusional and biochemical limitations. Greater *A*_n_ was found in lower‐latitude accessions native to climates with more frost‐free days, greater precipitation seasonality and lower temperature seasonality. As expected, water stress consistently impaired photosynthesis with the highest % reductions in lower‐latitude accessions (A3, A5 and A9), which had the highest *A*_n_ under well‐watered conditions. However, *A*_n_ for A3 and A5 remained among the highest under dehydration. *J. regia* accessions, which have leaf structural traits and biochemistry that enhance photosynthesis, could be used as commercial scions or breeding parents to enhance productivity.

## Summary Statement

*Juglans regia* accessions originating from lower latitudes exhibited enhanced photosynthetic capacity associated with increased gas‐phase diffusion and leaf nitrogen, and lower leaf mass and stomatal density. These accessions hold potential to improve productivity and stress tolerance for commercial production.

## 1 INTRODUCTION

Common walnut, *Juglans regia* L., is an important and widely grown agronomic species with major production areas concentrated in the northern hemisphere. Its natural range encompasses mountains from western China to central Asia (McGranahan & Leslie, 2009) and was extended by humans spreading the species throughout eastern and southwestern Europe from Central Asia (Leslie & McGranahan, 1998). Throughout its natural habitat, *J. regia* grows under a range of climatic conditions with mean monthly maximum temperatures ranging from −9 to +30°C and annual cumulative precipitation from 175 to 1150 mm during the growing season (Geospatial Data; Duke, 1978; FAO, 2021 [https://aquastat.fao.org/climate-information-tool/]; Figure S1). *J. regia* has been utilized to develop commercial scion cultivars (e.g., Chandler) and hybrid rootstocks with resistance to abiotic and biotic stresses (Kluepfel et al. 2015; Leslie, et al. 2015). Global walnut production is highly dependent on the limited genetic diversity of the commonly used scions. For example, only four scion cultivars account for ~80% of total yields. This results in orchard susceptibility to abiotic stresses, disease and pathogens. In California, the Chandler cultivar accounts for 50% of productive acreage (USDA‐NASS, 2020) but has limited capacity in dealing with high temperatures and water deficits, which can also increase susceptibility to plant pathogens and low‐quality kernel production (Grant & Shackel, 1998; Lampinen et al., 2005; Rosati et al., 2006). Wild germplasm collections can serve as a valuable resource to increase genetic variability to improve tolerance to abiotic and biotic stressors and crop productivity. The diverse *J. regia* collection at the U.S. department of agriculture, agricultural research service (USDA‐ARS) National Clonal Germplasm Repository (NCGR) located in Winters, CA, USA, holds such potential. However, to date, this collection has not been exploited to identify genotypes with increased abiotic stress tolerance and physiological traits related to enhanced yield.

Photosynthesis is a key determinant of crop productivity and positively related to biomass accumulation and yield production (Faralli & Lawson, 2020; Fischer et al., 1998; Kruger & Volin, 2006; Long et al. 2006; Simkin et al. 2019). Photosynthetic CO_2_ response curves (*A*_n_ *− C*_i_; net assimilation, *A*_n_ vs. CO_2_ inside the leaf, *C*_i_) can be used to assess the biochemical and diffusive limitations that determine photosynthetic rates (Long & Bernacchi, 2003; Sharkey 2016). The biochemical limitations are determined from the maximum carboxylation rate of Rubisco (*V*_cmax_), the maximum rate of electron transport (*J*_max_) and the maximum rate of triose phosphate use (TPU), all derived from *A*_n_ *− C*_i_ curves. When combined with chlorophyll fluorescence measurements, *A*_n_ *− C*_i_ curves can also provide information on diffusive limitations associated with mesophyll conductance (*g*_m_; Harley et al., 1992), which is a measure of the ease with which CO_2_ diffuses from the substomatal cavity to the site of carboxylation inside chloroplasts. Recently, these biochemical characteristics have been used to evaluate germplasm in crop breeding programmes (De Souza & Long, 2018; De Souza et al., 2020).

Identifying variation in diffusive limitations which are strongly linked to *g*_m_ and leaf structure can also be used in breeding programs to improve photosynthesis (Tomás et al., 2013; Tosens et al., 2012)*. g*_m_ involves a complex pathway and a series of resistances in both the gas and liquid phases (Flexas et al., 2008; Tosens & Laanisto, 2018), and is impacted by various leaf structural traits including intercellular airspace (IAS) volume (i.e., porosity), mesophyll surface area exposed to the IAS (*SA*_mes_/*V*_mes_), mesophyll cell diameter and density, and cell wall thickness (Evans, 2021; Flexas et al., 2008, 2012; Théroux‐Rancourt & Gilbert, 2017). Leaf structure of some species exhibits plasticity in response to the growth environment (Salk, 2012), resulting in functional variation, which can help optimize resource use (Muir et al., 2017; Wright et al., 2004). Any inherent variation in leaf structural and physiological traits, as a function of the habitat environment to which it has been adapted, may play an important role in regulating photosynthetic capacity in *J. regia* accessions. We also recently found that two *Juglans* spp. exhibit changes in mesophyll structure under dehydration associated with changes in cell volume, orientation and arrangement that increases porosity (Momayyezi et al., 2022). Desiccation influences CO_2_ diffusion and water relations as mesophyll cell turgor changes (Buckley et al., 2017; Scoffoni et al., 2014). How genotypic diversity in mesophyll cell packing and distribution across *J. regia* accessions may link with photosynthetic performance and susceptibility to drought is yet to be investigated. X‐ray microcomputed tomography (microCT) provides an in‐depth assessment of leaf mesophyll traits (i.e., porosity and tortuosity) and cells orientation and geometry (Earles et al., 2018, 2019; Lundgren & Fleming, 2020; Théroux‐Rancourt et al., 2020).

In this study, we combined gas exchange physiological analysis with microCT imaging of leaves to explore: (1) the photosynthetic capacity of numerous *J. regia* wild accessions originating from habitats with varied climatic conditions; (2) links between leaf structural diversity and physiological features that enhance photosynthetic capacity; and (3) whether genotypic differences hold up under water stress conditions. Based on our previous observations for two *Juglans* species (Momayyezi et al. 2022), we hypothesize that greater *A*_n_ will be associated with thicker leaves and higher mesophyll porosity and gas‐phase diffusion (*g*_IAS_; Han et al., 2019; Tomás et al., 2013). We also expect accessions originating from lower latitudes, characterized by warmer and wetter habitats, would exhibit higher inherent *A*_n_ to support growth through enhanced biochemical activities as predicted for plants under warmer conditions (Moore et al., 2021; Ruiz‐Vera et al., 2018). We anticipate that greater *A*_n_ would be concurrent with higher IAS and conductance as temperature and precipitation are known to strongly influence functional diversity of photosynthesis across species (Harrison et al., 2020; Ordonez & Svenning, 2017).

## 2 MATERIALS AND METHODS

**Figure 1:**
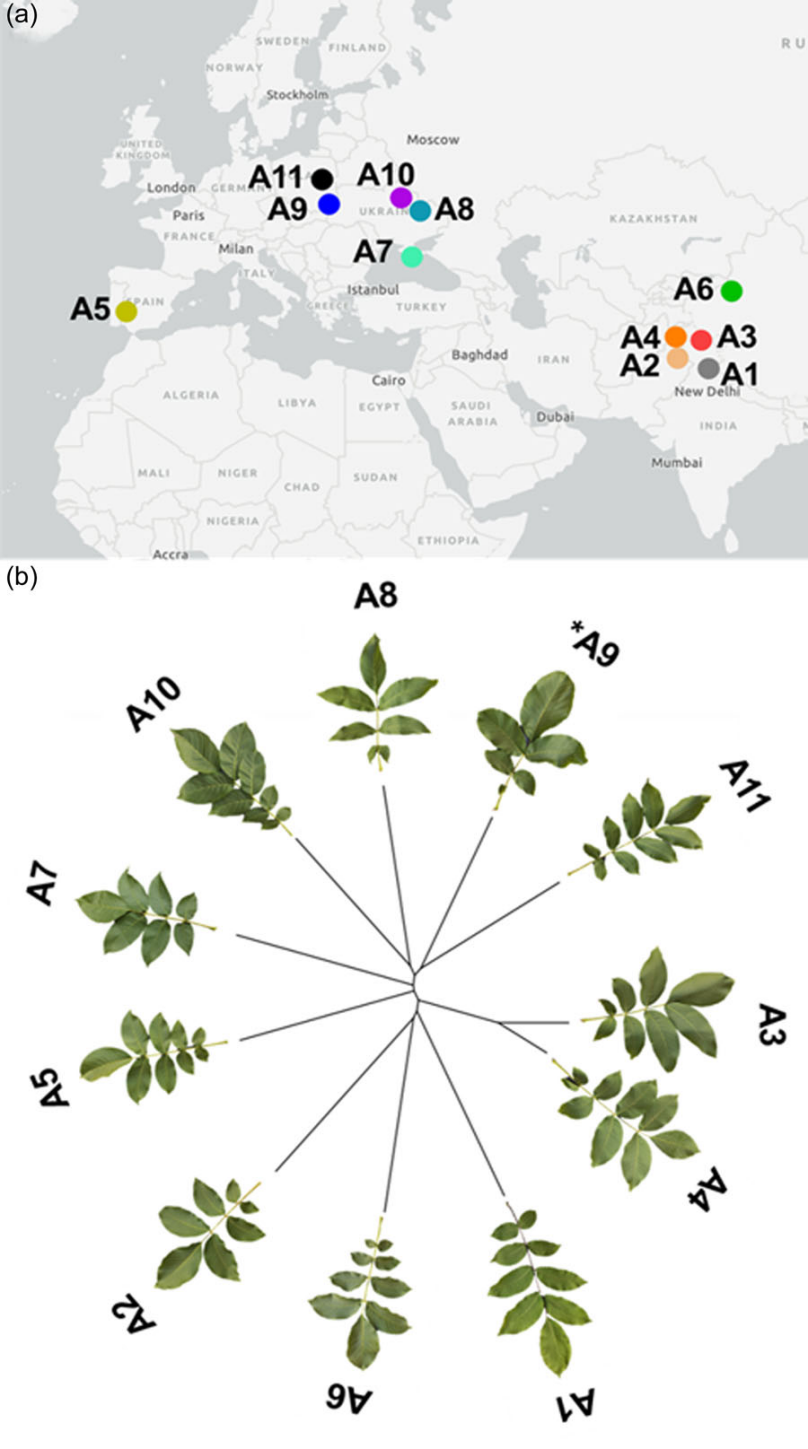
(a) Geographic distribution map for 11 *J. regia* accessions. Location data were found in the USDA‐ARS National Plant Germplasm System for individual accessions (Germplasm Resources Information Network, GRIN). (b) Unrooted neighbour‐joining tree for 11 *J. regia* accessions. Thirteen thousand three hundred and twenty polymorphic single nucleotide polymorphisms (SNPs) among these 11 trees were discovered by Illumina sequencing for the tip leaflet and used to construct NJ trees in R using the “phangorn” package. *Genotype data from C4 29, different individual but same accession as A9 (C4 28; see Table S1) was used, as data for original A9 was not available. [Color figure can be viewed at wileyonlinelibrary.com]

Stems were collected from 11 genetically unique *J. regia* accessions (Figure 1a,b) at the USDA‐ARS‐NCGR in Wolfskill Experimental Orchard, Winters, CA, USA, to use as scion and were grafted by Sierra Gold Nursery onto a commonly used commercial rootstock, RX1 (*Juglans microcarpa* × *J. regia*). We used a common rootstock to eliminate any own‐root effects and to simulate conditions for a commercial walnut orchard setting, where rootstocks are commonly used. The grafted saplings were repotted and transferred to the Armstrong lathe house facility at the University of California, Davis, in June 2019, and kept under natural light, temperature and relative humidity (~50%). The budburst for accessions occurred between April and May.

Measurements were initiated on nonstressed plants in August 2019 under well‐watered conditions for all accessions. The measurements on the same plants were repeated under dehydrated condition through a gradual dry down procedure using the methods as described by Knipfer et al. (2020). Briefly, water loss from pots via transpiration and soil evaporation were quantified during the experiment by weighing pots to calculate the required amount of water needed per pot under each treatment, daily. After completion of measurements in nonstressed conditions, water application was reduced to 75% of full irrigation during the first week and then to 50% of full irrigation in the second week of drying. This watering regime was then maintained for the dehydration treatment until completion of the experiment. Plants were maintained under ambient natural light with a ~15 h photoperiod during the experiment, maximum temperature of 35°C during day and minimum of 15°C during night, in 2.65 L pots containing a 40% pine bark, 40% sphagnum peat moss and 20% vermiculite, and a thin layer of the slow‐release fertilizer (Osmocote Smart‐Release Plus) was added to the top soil at the plantation time. The two irrigation treatments were maintained for ~2 weeks before the measurements.

### 2.1 Illumina sequencing and phylogeny tree construction

Genotyping‐by‐sequencing libraries were prepared by restricting genomic DNA with HindIII‐HF (NEB) with Ampure XP bead (Beckman‐Coulter) cleanup. Sequence data were obtained on an Illumina Hiseq 4000 instrument at the UC Davis Genome Center. Single nucleotide polymorphism (SNP) calling was performed using the TASSEL GBS pipeline (Glaubitz et al., 2014) with alignment to the Chandler genome (Marrano et al., 2020), resulting in 13,320 polymorphic SNPs. The phangorn package in R (R Core Team, 2017; Schliep et al., 2017) was used to construct a neighbour‐joining tree from the distance matrix.

### 2.2 Photosynthetic measurements

Net assimilation rate (*A*_n_), stomatal conductance (*g*_s_) and the IAS CO_2_ concentration (*C*_i_) were measured on one leaflet of the leaf from each accession in five replications using a LI‐COR 6800 system fitted with 6800‐01A fluorometer. To provide a general leaf developmental assessment before the measurements, the leaf plastochrone index (LPI) was calculated as described by Erickson & Michelini (1957). The 4th or 5th leaflet was counted down from the tip leaflet on the leaves at LPI between 5 and 6. All measurements were done under photosynthetic photon flux density (PPFD) of 1500 µmol m^−2^ s^−1^ (10% blue vs. 90% red), a saturating PPFD for all accessions (Figure S2), chamber temperature at 25°C, ambient chamber CO_2_ concentration (*C*_a_) at 400 (µmol mol^−1^), flow rate at 500 (µmol air s^−1^) and vapour pressure deficit between 1.5 and 2.0 kPa. To calculate the intrinsic water use efficiency (WUE_i_), *A*_n_ was divided by *g*_s_ for each accession in five replications under both well‐watered and dehydrated conditions. The 6 cm^2^ round fluorometer gasket set was filled fully with the leaflet area and was changed frequently between the measurements, along with running corrections suggested by the manufacturer to reduce errors of CO_2_ and H_2_O leakage through the gasket. To obtain the maximum quantum yield of photosystem II, all leaflets were dark adapted for 20 min before all other measurements. The quantum yield of photosystem II (Φ_PSII_) under actinic light was obtained by application of saturating multiphase flashes (>8000 µmol m^−2^ s^−1^) as in Genty et al. (1989).

### 2.3 Calculation of *g*_m_ by chlorophyll fluorescence and of CO_2_ concentration in the chloroplast (*C*_c_)

The “variable *J* method” was used to estimate *g*_m_ based on calculation of electron transport rate (*J*_flu_) from measurements of chlorophyll fluorescence (Bongi & Loreto, 1989; Harley et al., 1992):

(1) $$J_{\mathrm{flu}}=\Phi_{\text{PSII}}\times \text{PPFD}\times \alpha \times \beta$$

where *β* (0.5 for C_3_ plants) is the fraction of absorbed quanta reaching photosystem II (Bernacchi et al., 2002). The leaf absorbance, *α*, was previously measured for *Juglans* species to be 85.3% based on the average value (±0.2 SE) in all individuals using an ASD Fieldspec spectroradiometer (ViewSpec Pro, ASD Inc.; Momayyezi et al., 2022). *g*_m_ was given by (Harley et al., 1992):

(2) $$g_{m}=A_{n}/\left[C_{i}-\left(\frac{\Gamma^{*}\left(J_{\mathrm{flu}}+8\left(A_{n}+R_{d}\right)\right)}{J_{\mathrm{flu}}-4\left(A_{n}+R_{d}\right)}\right)\right]$$

where *R*_d_ is the nonphotorespiratory respiration rate in the light and Γ* is the chloroplast CO_2_ photocompensation point. Γ* was assumed to equal the intercellular CO_2_ photocompensation point (*C*_i_*) per Gilbert et al. (2012). We used previously reported *R*_d_ ± SE (0.73 ± 0.08 µmol m^−2^ s^−1^) and *C*_i_* ± SE (38.18 ± 0.47 µmol mol^−1^), which were found using the Laisk method (Laisk, 1977, in Gilbert et al., 2012) as the point of intersection of the linear portion of averaged four sets of *A*_n_ − *C*_i_ curves obtained at three irradiances (100, 200 and 500 µmol m^−2^ s^−1^) and 13 CO_2_ concentrations (35, 40, 50, 60, 70, 80, 90, 100, 110, 120, 140, 160 and 180 µmol mol^−1^). We used this approach previously for two *Juglans* species with contrasting leaf anatomy (Momayyezi et al., 2022) and applied it to the 11 *J. regia* accessions in this study. We assumed any potential small changes in *C*_i_* and *R*_d_ under dehydration will be negligible, and the same values measured for *Juglans* species under well‐watered conditions were used to estimate *g*_m_ under dehydration. Having obtained *g*_m_ by the chlorophyll fluorescence method, the CO_2_ concentration in the chloroplast (*C*_c_) was found according to Harley et al. (1992):

(3) $$C_{c}=C_{i}-\frac{A_{n}}{g_{m}}$$

### 2.4 *A*_n_ − *C*_i_ curves

To better understand photosynthetic responses, we constructed CO_2_ response (*A*_n_ − *C*_i_) curves for each accession using five replications at saturating PPFD (1500 µmol m^−2^ s^−1^) under the following sample CO_2_ concentration: 400, 50, 80, 100, 150, 200, 400, 600, 800, 1000, 1200, 1500 p.p.m. under well‐watered and dehydrated conditions. Before running *A*_n_ − *C*_i_ curves, the seal of the fluorometer chamber was tested for CO_2_ leakage by running a full curve at different *C*_a_ concentration in empty cuvette and the measurements were corrected using the Licor's equation (Bernacchi et al., 2002; Flexas et al., 2007). The *A*_n_ and corresponding *C*_i_ values for each accession in five replications were introduced to the Sharkey's fitting calculator version 2.0 (Sharkey 2016), a Farquhar‐von Caemmerer‐Berry‐based model to estimate *V*_cmax_, *J*_max_ and *g*_m_ using differences in CO_2_ partial pressure between *C*_i_ and *C*_c_ and predict assimilation rates (*A*) at the limiting states of Rubisco, RuBP‐regeneration and TPU (Sharkey et al., 2007). We used photosynthesis at the TPU state to set the maximum assimilation rate (*A*_max_) at saturating CO_2_ as in Sharkey et al. (2007). *g*_m_ obtained from the chlorophyll fluorescence method was verified against *g*_m_ found using *A*_n_ − *C*_i_ method (Figure S3). *g*_m_ obtained from the chlorophyll fluorescence method was used to present correlations, prepare figures and tables and make statistical comparisons for each accession under both well‐watered and dehydrated conditions.

### 2.5 Leaflet water potentials

Leaflet water potential (Ψ_leaflet_) was measured using a pressure chamber (PMS Instrument Company, Model 1505D) immediately after gas exchange measurements between 10 AM to 12 PM (Williams & Araujo, 2002). A leaflet directly opposite the one used for gas‐exchange measurements was used to measure leaflet water potential from each accession at five replications. Each leaflet was cut at petiolule base and bagged for 10–15 min to allow equilibration within the leaflet. Then, the petiolule was placed inside the pressure chamber gasket. Chamber pressure was increased slowly until the balancing pressure was reached.

### 2.6 Leaf mass and nitrogen content per area

Immediately after gas‐exchange measurements, the leaflet adjacent to the one used for gas exchange was punched and collected discs were dried at 72°C oven for 48 h. Using the leaflet discs area and dry weight, leaf mass per area (LMA) was calculated for each accession using five replications. Later, the dried samples were ground and 4.5–5.0 mg of them were encapsulated in tin cups and sent to the Stable Isotope Facility, at University of California Davis for total nitrogen content elemental analysis. Using LMA data, the nitrogen content results per unit mass were converted to leaf nitrogen content per unit area (Leaf N).

### 2.7 X‐ray microCT imaging

Leaves from each accession and treatment in five replications were scanned using X‐ray microCT at beamline 8.3.2 at the Advanced Light Source (ALS) in Lawrence Berkeley National Laboratory, Berkeley, CA, USA. The same leaflet samples used for gas exchange were collected from the plants, bagged and placed in a cooler at room temperature an hour before scanning in ALS. Leaves from the well‐watered conditions were collected and scanned in September 2019. After the plants went through the dehydration process, leaves were similarly collected and scanned at ALS in October 2019. A single piece of 3 mm‐wide and 7 mm‐long was taken from middle of the leaflet lamina from each plant and enclosed between two pieces of Kapton tape to prevent desiccation of the tissue and sample movement during the scanning. Samples were placed inside the end of a pipette tip and scanned under a continuous tomography mode at 23 keV using ×10 objective lens with a pixel resolution of 0.65 μm. Raw tomographic data were reconstructed using TomoPy (Gürsoy et al., 2014) through both gridrec and phase‐retrieval reconstruction (Davis et al., 1995; Dowd et al., 1999).

### 2.8 Mesophyll surface area, porosity, tortuosity and lateral path lengthening

Mesophyll porosity, *θ*_IAS_ (m^3^ m^−3^) was calculated as the IAS volume as a fraction of the total mesophyll volume as described by Théroux‐Rancourt et al. (2017). The IAS volume (*V*_IAS_) to mesophyll cell volume (*V*_mes‐cell_) ratio and the mesophyll surface area exposed to the IAS (*SA*_mes_) per mesophyll volume (*V*_mes_) were calculated as *V*_IAS_/*V*_mes‐cell_ (m^3^ m^−3^) and *SA*_mes_/*V*_mes_ (μm^2^ μm^−3^), respectively.

The tortuosity factor, *τ* (m^2^ m^−2^), was the diffusive path length within the IAS (i.e., the actual path from the stomate to a cell surface; geodesic distance [*L*_geo_]) to the straight path length without any physical obstacles to diffusion between the stomate and the cell surface (Euclidean distance, *L*_Euc_):

(4) $$\tau ={\left(\frac{L_{\mathrm{geo}}}{L_{\mathrm{Euc}}}\right)}^{2}$$

as described in Earles et al., (2018). The *L*_geo_ and *L*_Euc_ were mapped and quantified for all voxels along the mesophyll surface and *τ* was calculated for the whole three‐dimensional (3D) image array as in Earles et al. (2018). Then, leaf‐level tortuosity (*τ*_leaf_) was calculated as the mean of *τ* values at the edge of mesophyll cells. The lateral path lengthening *λ* (m m^−1^) was calculated using *L*_Euc_ and a second distance map as described by Earles et al. (2018) to measure the shortest unobstructed distance in a straight line between the abaxial epidermis and all points along the mesophyll surface *L*_epi_ (Legland et al., 2016):

(5) $$\lambda =\frac{L_{\mathrm{Euc}}}{L_{\mathrm{epi}}}$$

Similarly, leaf‐level lateral path lengthening (*λ*_leaf_) was then calculated as the mean of *λ* values at the edge of mesophyll cells.

Vein volume relative to the leaf volume ratio (*V*_vein_/*V*_leaf_, m^3^ m^−3^) was calculated as a fraction of total leaf volume as described by Trueba et al. (2022) from the same scans used for *θ*_IAS_, *τ* and λ_leaf_ calculations.

### 2.9 IAS conductance and stomatal density

The *τ*_leaf_, *λ*_leaf_ and *θ*_IAS_ were used to calculate leaf‐level IAS conductance (*g*_IAS_), where *D*_m_ is the diffusivity of CO_2_ in air (m^2^ s^−1^). Diffusion path length in gas phase was equal to half of the mesophyll thickness (*L*_mes_) for hypostomatous leaves (Earles et al., 2018; Niinemets & Reichstein, 2003; Tomás et al., 2013):

(6) $$g_{\mathrm{IAS}}=\frac{\theta_{\mathrm{IAS}}\;D_{m}}{{0.5L}_{\mathrm{mes}}\tau_{\mathrm{leaf}}\lambda_{\mathrm{leaf}}}$$

To verify autosegmentation and IAS trait estimation by random forest model, a PyTorch implementation of a fully convolutional network model with a ResNet‐101 backbone was used for the semantic segmentation of the leaf image data with cloud‐based resources in Google Colab. For training, we used a binary cross‐entropy loss function, an Adam optimizer for stochastic optimization with a learning rate of 0.001, a scaling factor of 1 to avoid small feature loss in the training images and a batch size of 1 to accommodate the GPU limitations in Google Colab. Output results were comparable to those generated on the same image sets with a workflow developed by Théroux‐Rancourt et al. (2020) using a random forest model for semantic segmentation of leaf tissues. The output was used to validate tissue surface area and volume determination and 3D leaf projection (Figure S4).

To quantify the stomatal density, the grid reconstructions were used at the paradermal direction. The stomata density and size were measured in 0.04 mm^2^ of leaflet area for all accessions (*n* = 5).

### 2.10 Climatic data for accessions' native habitats

Coordinates for each accession's native habitats were extracted from the USDA‐ARS Germplasm Resources Information Network database (https://npgsweb.ars-grin.gov/gringlobal/search). Temperature and precipitation data were obtained from FAO climate information tools (https://aquastat.fao.org/climate-information-tool/) for each of these native habitat locations (Figure S1). The temperature and precipitation seasonality were calculated based on the ratio of the SD for monthly mean temperature or precipitation to the monthly mean temperature or precipitation (known as the coefficient of variation) and multiplied by 100. The temperature or precipitation values for each month were averaged and the SD was calculated using the mean of the 12 months (Donnell & Ignizio, 2012).

### 2.11 Statistics

Linear regression and Pearson correlation coefficients were used to examine relationships between latitude, temperature and precipitation seasonality, frost‐free days and *A*_n_, *A*_max_, *g*_s_, *g*_m_, *g*_IAS_, *L*_leaf_, *θ*_IAS_, *λ*_leaf_, *τ*, *V*_vein_/*V*_leaf_, WUE_i_, Leaf N, LMA, *J*_max_, *V*_cmax_ and Ψ_leaflet_ using GraphPad prism 9 software (GraphPad Software, Inc.). Paired *t*‐test was used to check for systematic differences between the chlorophyll fluorescence and *A*_n_ − *C*_i_ curve methods for estimating *g*_m_ and *C*_c_. Mixed linear models were used to compare relative changes in percent for *A*_n_, *g*_m_, *g*_s_, *L*_leaf_, *g*_IAS_, *θ*_IAS_ and Ψ_leaflet_ under dehydration for all accessions using SAS 9.4 (SAS Institute Inc., 2013). The *p* required for significance (0.002) was adjusted by dividing *α* (0.05) by the number of comparisons per test (25 here). Logarithm or squared transformations were performed to meet normality and equal variance assumptions where needed.

## 3 RESULTS

Inherent differences in photosynthetic capacity were found among the accessions; *J. regia* accessions 3, 5 and 9 showed the highest photosynthetic capacity, as measured by *A*_max_ (26.3, 25.6, 27.5 μmol CO_2_ m^−2^ s^−1^, respectively). Higher *A*_max_ in these accessions was linked to greater maximum carboxylation rate (*V*_cmax_, *R* ^2^ = 0.81, *p* < 0.001) and maximum electron transport rate (*J*_max_, *R* ^2^ = 0.67, *p* = 0.002; Figurse 3 and S6). Leaves with higher *A*_max_ had thicker leaves (*L*_leaf_, *p* = 0.013) with greater mesophyll porosity (*θ*_IAS_, *p* = 0.049) and leaf nitrogen (Leaf N, *p* = 0.044; Figure S6).

**Figure 2:**
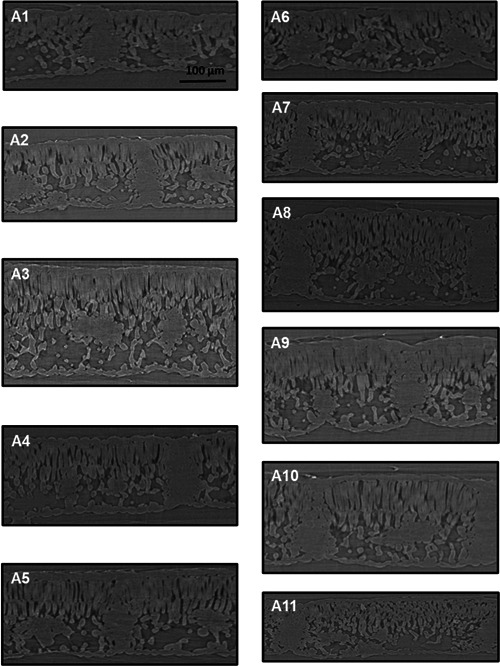
Leaf cross sections from representative scans for 11 *J. regia* accessions under well‐watered condition obtained using X‐ray microcomputed tomography.

**Figure 3:**
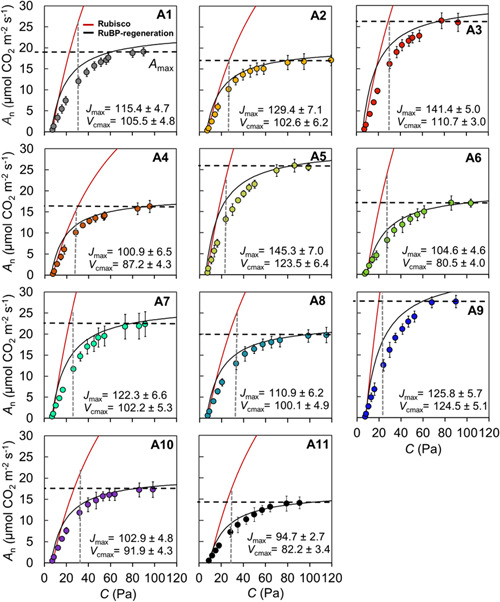
Photosynthetic CO_2_ response curves were constructed using Sharkey's fitting calculator version 2.0 (Sharkey 2016), averaged for 5 replications in 11 *J. regia* accessions under well‐watered condition. *A* − *C*_i_ curves are shown with coloured circles and error bars measured directly. *A* − *C*_c_ curves were used to generate *V*_cmax_ and *J*_max_, and averaged over five replications for each accession (±SE, *n* = 5). Black dashed horizontal line indicates assimilation rate at saturating CO_2_ (*A*_max_) at triose phosphate use (TPU) limitation state, and Rubisco and RuBP regeneration limitations are indicated for each accession by red and black curves, respectively. Colour scheme is consistent with accession numbers presented in Figure 3 and in Table 1. Dashed vertical grey lines on each plot represent the *C*_i_ at ambient CO_2_ of 40.4 Pa, which represents the limitation of *g*_m_ in comparison with the value of the *A* − *C*_c_ curve (i.e., the point where *C*_i_ and *C*_c_ would be equal). [Color figure can be viewed at wileyonlinelibrary.com]

**Figure 4:**
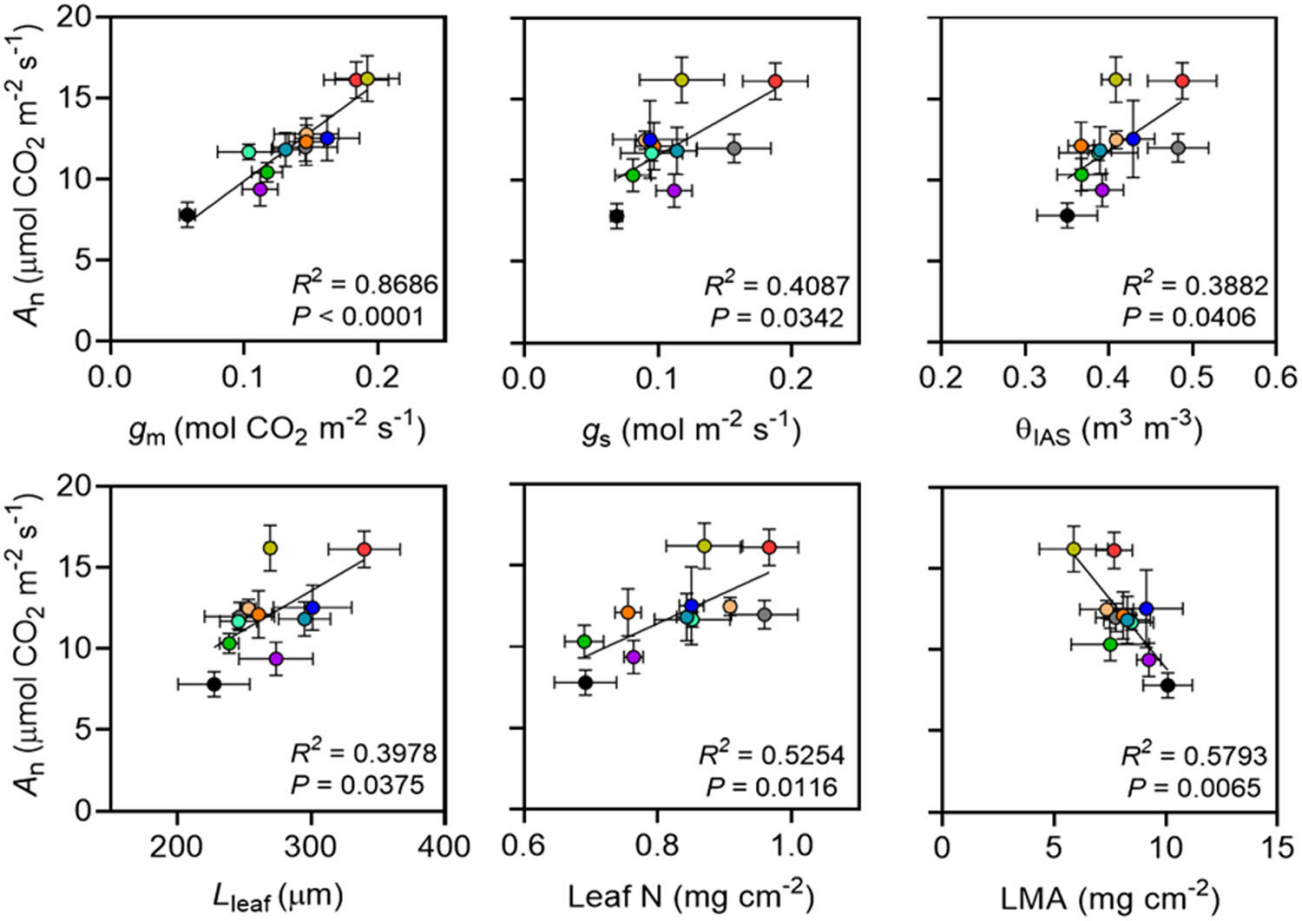
Net assimilation rate (*A*_n_, μmol CO_2_ m^−2^ s^−1^) relationship with mesophyll conductance obtained from chlorophyll fluorescence method (*g*_m_, mol CO_2_ m^−2^ s^−1^), stomatal conductance (*g*_s_, mol m^−2^ s^−1^), mesophyll porosity (*θ*_IAS_, m^3^ m^−3^), leaf thickness (*L*_leaf_, μm), leaf nitrogen per unit area (Leaf N, mg cm^−2^) and leaf mass per unit area (LMA, mgcm^−2^) in 11 *J. regia* accessions using mean values (±SE, *n* = 5). *A*_n_ was extracted from *A*_n_ − *C*_i_ curves at *C*_a_ of 40.4 Pa. Colour scheme is consistent with accession numbers presented in Figure 3 and in Table 1. [Color figure can be viewed at wileyonlinelibrary.com]

Similarly, *A*_n_ was extracted from *A*_n_ − *C*_i_ curves at *C*_a_ of 40.4 (Pa) and was positively correlated with chlorophyll fluorescence *g*_m_ (*p* < 0.0001) and *g*_s_ (*p* = 0.034; Figure 4) across the accessions. Similar to *A*_max_, leaves with greater *A*_n_ had thicker leaves (*L*_leaf_, *p* = 0.037) and mesophyll (*L*_mes_, *p* = 0.05; data not presented), higher mesophyll porosity (*θ*_IAS_, *p* = 0.041) and nitrogen content per unit area (Leaf N, *p* = 0.012) but less leaf mass per unit area (LMA, *p* = 0.007; Figure 4). Increased *A*_n_ was not significantly related with lateral path lengthening (*λ*_leaf_, *p* = 0.091) nor tortuosity (*τ*_leaf_, *p* > 0.1; data not presented). Leaves with greater *g*_m_ exhibited greater *θ*_IAS_ (*R* ^2^ = 0.42; *p* = 0.030), higher mesophyll thickness (*p* = 0.034) and had lower *λ*_leaf_ (*p* = 0.031; Figure S7). High‐porosity leaves had lower *V*_vein_/*V*_leaf_ (*p* = 0.049) and WUE_i_ (*p* = 0.047; Figure S7), and exhibited greater *g*_s_ (*p* = 0.0003), concurrent with lower stomatal density (*R* ^2^ = 0.38; *p* = 0.042). Across accessions, leaflet water potential (Ψ_leaflet_) was positively related to both *θ*_IAS_ (*p* = 0.024) and *g*_s_ (*p* = 0.028).

### 3.1 Climate‐driven photosynthetic capacity

**Figure 5:**
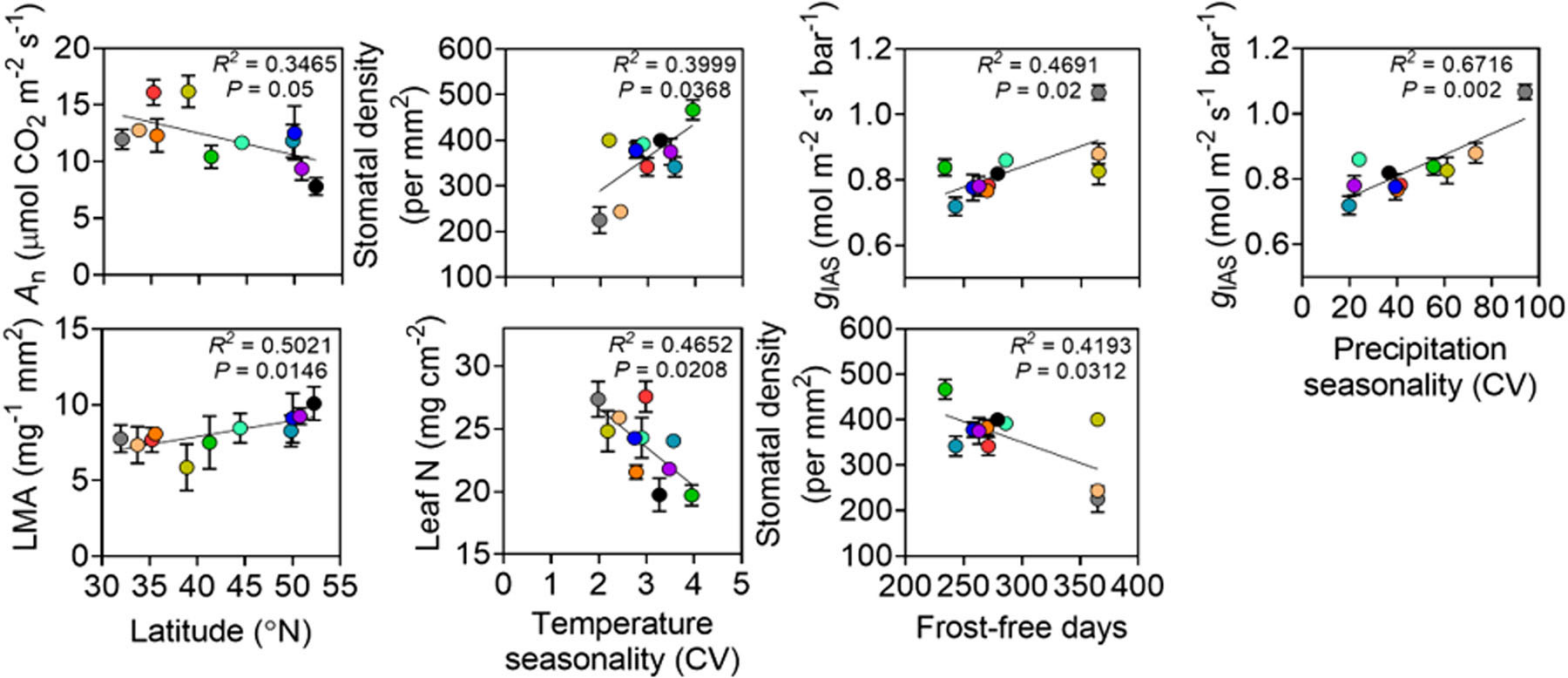
Relationship between net assimilation rate (*A*_n_, μmol CO_2_ m^−2^ s^−1^), intercellular airspace (IAS) conductance (*g*_IAS_, mol O_2_ m^−2^ s^−1^ bar^−^^1^), stomatal density (mm^−2^), leaf mass per unit area (LMA, mg cm^−2^) and leaf nitrogen per unit area (Leaf N, mg cm^−2^) using mean values (±SE, *n* = 5) and latitude (°N), temperature seasonality (coefficient of variation [CV]), precipitation seasonality (CV), and frost‐free days in habitats for 11 *J. regia* accessions (see Figure S5 for the full trait correlations). [Color figure can be viewed at wileyonlinelibrary.com]

Photosynthetic capacity and associated leaf physiological and anatomical characteristics for the accessions were associated with the climatic conditions in native habitats. Accessions from habitats with lower temperature seasonality in lower latitudes had higher *g*_ias_ and Leaf N (*p* ≤ 0.05), which may support higher their *A*_n_ through greater *g*_m_ and *θ*_IAS_ (Figure 5; *p* = 0.05; Figure S5). Despite nonsignificant relationships, parallel decreases in *g*_m_, *g*_IAS_, Leaf N and *θ*_IAS_ (*p* > 0.05; Figure S5) may suggest a pattern for decline in mesophyll CO_2_ diffusion rate with latitude, while they accumulate more LMA (*p* = 0.015; Figure 5). Higher variability in precipitation seasonality and more frost‐free days were significantly related to increased *g*_IAS_ concurrent with lower stomatal density (Figure 5). Decreases in LMA and frost‐free days were associated with increases in Leaf N (Figure 5).

### 3.2 Responses under dehydration

As expected, dehydration impaired photosynthesis and altered leaf structure with reduced *g*_s_, *g*_m_ and *L*_leaf_ and increased *θ*_IAS_ and *g*_IAS_ in all accessions. The percent reduction in *A*_n_ was significantly correlated with percent reductions in *g*_s_ (*p* = 0.007), *g*_m_ (*p* = 0.002) and Ψ_leaflet_ (*p* = 0.002) under dehydration (Table 1). Accessions A3, A5 and A9, which had the highest *A*_n_ and *A*_max_ under well‐watered conditions, exhibited the greatest percent reductions amongst accessions in these parameters under drought stress (i.e., >50% reduction for all 3; Table 1). However, the absolute values of *A*_n_ for accessions A3 and A5 were not significantly lower than other accessions under dehydration, whereas they were among the lowest for A9 under dehydration (Table S2). The reduction in *L*_leaf_ was linked with decreases in *g*_ias_ (*p* = 0.02) and *g*_m_ (*p* = 0.08). The concurrent reduction in Ψ_leaflet_ was significantly correlated with percent decline in *g*_s_ (*p* = 0.004; Table 1). Under dehydration, absolute *A*_n_, *g*_m_, *θ*_IAS_, *g*_IAS_ and Leaf N remained negatively correlated with latitude (*p* < .05, Table 2).

**Table 1:** Percent change in physiological and anatomical variables under dehydration relative to the well‐watered condition

Accession #	*A*_n_	*g*_s_	*g*_m_	Ψ_leaflet_	*L*_leaf_	*θ*_IAS_	*g*_IAS_
A 1 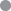	−20.8 ± 0.8 (A2,3,4,5,6,8,9,10,11)	−26.0 ± 3.4 (A2,3,4,5,9,10,11)	−36.4 ± 2.4 (A2,3,4,5,6,7,8,9,10,11)	−39.8 ± 2.7 (A3,5,9)	−8.9 ± 0.8 n.s.	+8.4 ± 1.9 (A3)	+16.5 ± 2.2 (A2,3,6,9)
A 2 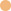	−36.2 ± 0.8 (A1,3,5,7,9,10)	−58.3 ± 0.8 (A1,4,6,7,8,9,11)	−53.4 ± 1.9 (A1,5,7)	−49.4 ± 2.3 (A3)	−11.7 ± 1.2 n.s.	+17.0 ± 1.9 (A8,10)	+33.3 ± 1.4 (A1,4,7,8,10,11)
A 3 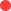	−50.8 ± 1.6 (A1,2,4,6,7,8)	−66.8 ± 2.1 (A1,4,6,7,8,11)	−54.1 ± 2.6 (A1,5,7)	−66.5 ± 3.8 (A1,2,4,6,7,8,11)	−9.1 ± 0.9 n.s.	+21.8 ± 2.6 (A1,5,6,7,8,10,11)	+35.4 ± 2.5 (A1,4,7,8,10,11)
A 4 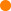	−36.9 ± 1.1 (A1,3,5,7,9,10)	−44.5 ± 1.1 (A1,2,3,5,6,7,8,9,10,11)	−61.1 ± 1.9 (A1,5,7,11)	−37.8 ± 3.3 (A3,5,9,10)	−7.6 ± 0.8 (A6)	+15.2 ± 1.1 (A8,10)	+21.6 ± 1.9 (A2,3)
A 5 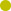	−56.7 ± 2.2 (A1,2,4,6,7,8,10,11)	−66.7 ± 1.8 (A1,4,6,7,8)	−77.5 ± 2.5 (A1,2,3,4,6,7,8,9,10,11)	−60.9 ± 3.6 (A1,4,6,7,8)	−11.5 ± 1.2 n.s.	+11.3 ± 1.2 (A3)	+24.8 ± 2.1 (A7,10)
A 6 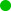	−35.6 ± 0.8 (A1,3,5,7,9,10)	−34.6 ± 0.9 (A2,3,4,5,7,9,10,11)	−60.7 ± 0.7 (A1,5,7,11)	−41.3 ± 2.6 (A3,5,9)	−13.0 ± 1.1 (A4,7,10)	+12.4 ± 2.8 (A3)	+31.2 ± 3.2 (A1,7,10,11)
A 7 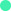	−19.9 ± 1.1 (A2,3,4,5,6,8,9,10,11)	−22.3 ± 2.3 (A2,3,4,5,6,8,9,10,11)	−20.2 ± 2.6 (A1,2,3,4,5,6,8,9,10,11)	−39.7 ± 4.6 (A3,5,9)	−6.9 ± 0.9 (A6)	+10.7 ± 1.5 (A3)	+13.2 ± 1.1 (A2,3,5,6,9)
A 8 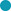	−40.7 ± 1.5 (A1,3,5,7,9)	−32.1 ± 1.6 (A2,3,4,5,7,9,10,11)	−57.8 ± 1.2 (A1,5,7)	−40.6 ± 2.8 (A3,5,9)	−9.3 ± 0.9 n.s.	+5.6 ± 1.2 (A2,3,4,9)	+20.8 ± 2.3 (A2,3)
A 9 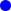	−52.2 ± 1.1 (A1,2,4,6,7,8,10,11)	−71.2 ± 1.8 (A1,2,4,6,7,8)	−61.1 ± 1.4 (A1,5,7,11)	−59.5 ± 3.2 (A1,4,6,7,8)	−8.7 ± 0.9 n.s.	+15.5 ± 0.9 (A8,10)	+28.5 ± 3.6 (A1,7,10)
A 10 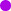	−44.9 ± 1.2 (A1,2,4,5,6,7,9,11)	−66.3 ± 1.3 (A1,4,6,7,8)	−50.2 ± 2.2 (A1,5,7)	−54.9 ± 3.1 (A4)	−7.4 ± 0.91 (A6)	+5.6 ± 1.3 (A2,3,4,9)	+12.5 ± 1.0 (A2,3,5,6)
A 11 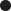	−35.6 ± 0.8 (A1,5,7,9,10)	−75.8 ± 4.2 (A1,2,3,4,6,7,8)	−49.4 ± 3.3 (A1,4,5,6,7,9)	−46.5 ± 1.8 (A3)	−8.3 ± 0.9 n.s.	+12.1± 2.3 (A3)	+19.4 ± 1.8 (A2,3,6)
*p*	<0.002	<0.002	<0.002	<0.002	<0.002	<0.002	<0.002

*Note*: Different accessions numbers are used to show significant differences under dehydration (treatment effect) from each other using mean values (±SE) over five replications at *p* < 0.002.

Abbreviations: *A*_n_, net assimilation rate (µmol CO_2_ m^−2^ s^−1^); *g*_s_, stomatal conductance (mol  m^−2^ s^−1^); *g*_m_, mesophyll conductance obtained from chlorophyll fluorescence method (mol CO_2_ m^−2^ s^−1^); Ψ_leaflet_, leaflet water potential (MPa); *L*_leaf_, leaf thickness (μm); *θ*_IAS_, mesophyll porosity (m^3^ m^−3^); *g*_IAS_, intercellular airspace conductance (mol m^−2^ s^−1^ bar^−1^).

**Table 2:** Pearson correlation coefficients between the absolute values

	Latitude (°N)	Temperature seasonality (CV)	Precipitation seasonality (CV)	Frost‐free days
*A*_n_	−0.728*	−0.541	0.447	0.553
*g*_m_‐ fluorescence	**−0.675**	−0.475	0.311	0.538
*θ*_IAS_	**−0.603**	−0.474	0.483	0.420
*g*_IAS_	−0.712*	−0.435	0.856*	0.582
Leaf N	**−0.603**	−0.083	0.372	0.01

*Note*: Pearson correlation coefficients between the absolute values of the physiological and anatomical variables and climatic data for 11 *J. regia* accessions under dehydration treatment using mean values (±SE, *n* = 5). Bold indicates significance at *p* < 0.05 and * indicates significance after Bonferroni corrections (*p* < 0.0025).

Abbreviations: *A*_n_, net assimilation; CV, coefficient of variation; IAS, intercellular airspace.

## 4 DISCUSSION

### 4.1 Photosynthetic capacity, mesophyll anatomy, and CO_2_ diffusion

Diverse accessions of *J. regia*, native to various habitats with different temperature and precipitation patterns, exhibited variable photosynthetic capacity. Three accessions (A3, A5 and A9) exhibited significantly higher *A*_n_, *A*_max_ and CO_2_ diffusion capacity under the well‐watered condition with the highest *V*_cmax_ and *J*_max_. Greater photosynthetic capacity is typically linked to carboxylation capacity via increased Rubisco protein abundance and activity (Hikosaka & Shigeno, 2009; von Caemmerer & Farquhar, 1981; Kattge et al., 2011).

Higher photosynthetic capacity was strongly linked to leaf thickness and mesophyll structure and supported by higher Leaf N. As expected, *J. regia* accessions with higher *A*_n_ had thicker leaves and exhibited greater *θ*_IAS_ and *g*_m_ (Figure 4). This agrees with previous findings for *J. regia* cv. Chandler (Momayyezi et al., 2022). *J. regia* accessions with higher *A*_n_ had greater *A*_max_, *V*_cmax_ and Leaf N, suggesting higher carboxylation capacity and performance (Figures 3 and S6). Although previous work reported a positive relationship between *A*_n_ with LMA (i.e. across *Quercus ilex* provenances; Peguero‐Pina et al., 2017), we found *J. regia* accessions exhibited greater *A*_n_ with lower LMA. Increasing cell density would reduce mesophyll surface area exposed to IAS as a result of high cell packing and could also be impacted by cell wall thickness (Niinemets et al., 2009a; Tomás et al., 2013; Tosens et al., 2012). A more porous mesophyll and thicker leaves with shorter *λ*_leaf_ (Figure S7), contributed to higher *A*_max_ across *J. regia* accessions (Figure S6), highlighting the fact that thickness and cell density may not change in the same direction (Niinemets, 1999; Syvertsen et al., 1995). Increases in leaf porosity are known to reduce diffusive resistance and lateral path lengthening in other species (Earles et al., 2018). Additionally, leaf mesophyll geometry and IAS are known to impact stomatal patterning, photosynthetic capacity and conductances (Baillie & Fleming 2020; Lehmeier et al., 2017; Lundgren et al., 2019); *J. regia* accessions with greater porosity had fewer but larger stomata with significantly greater *g*_s_.

Leaf anatomy also plays an important role in biophysical coordination between CO_2_ diffusion and leaf hydraulics (Boyce et al., 2009; Lehmeier et al., 2017; Fulton et al., 2017). Similar to findings from Trueba et al. (2022), *J. regia* accessions with greater porosity had less *V*_vein_/*V*_leaf_ and lower WUE_i_ (Figure S7) associated with higher *g*_s_. More extensive vasculature, including greater bundle sheath extensions, may improve WUE_i_ by improving connections between the vascular tissue and epidermis for stomatal regulation and water supply to replace losses due to transpiration (Brodribb et al., 2007; Zwieniecki et al., 2007).

### 4.2 Climatic variables and inherent functional diversity

We found that inherent differences in the photosynthetic activity of *J. regia* accessions is associated with climatic conditions in their native habitat (Figures 5 and S5). Other studies have shown that leaf structure and function are strongly related to the environment of a species' native habitat (Li et al., 2018; Reich 2014). Higher precipitation seasonality, concurrent with more frost‐free days in lower latitudes, was associated with higher *A*_n_ through increased *g*_m_ and *g*_IAS_. This can be due to increased allocation of Leaf N toward the dynamic biochemical activity rather than more static aspects of the mesophyll (e.g., wall thickness and mesophyll surface area; Evans, 2021; Terashima et al., 2006; Tosens et al., 2012). As discussed by He et al. (2018a), changes in leaf anatomy (i.e., leaf and epidermis thickness, and the ratio of spongy to palisade mesophyll) as a function of latitude are mainly driven by variability in precipitation and temperature. *J. regia* accessions from habitats from lower latitudes with warmer growing season and lower temperature seasonality had leaves with greater *g*_IAS_ and tortuosity. Greater precipitation during the warmest annual quarter (between June and August), when *J. regia* has the highest water demand for growth and fruit development, was associated with increased *g*_IAS_ (*p* = 0.02) and reduced SD (*p* < 0.001), suggesting a potential positive impact of irrigation on leaf performance by improving CO_2_ diffusion. The accessions with lower diffusion resistance (e.g., more porous leaves) benefit from a higher CO_2_ diffusion capacity, and exhibit inherently higher performance and biochemical activities (i.e., *V*_cmax_) under the common garden conditions of the experiment. Comparing *A* − *C*_i_ and *A* − *C*_c_ curves provides insights into the limitations imposed by mesophyll *g*_m_ on assimilation rate associated with the drawdown of CO_2_ from IAS (*C*_i_) to the chloroplast (*C*_c_; Figure 3). *J. regia* accessions from lower latitude exhibit enhanced performance through increasing *V*_cmax_ at low CO_2_ concentrations, found by the net assimilation rate response to *C*_c_ (*A* − *C*_c_ curve), when Rubisco is limiting *A* (Figure 3). That was in line with greater leaf nitrogen content for those accessions (Figure 4) and more investment toward Rubisco activity (Niinemets et al., 2009b; Sharkey et al., 2007; Warren & Adams, 2004), enhancing their *A*_n_ and CO_2_ diffusion capacity at lower CO_2_ concentrations (Figure 4). Greater maximum electron transport rate (*J*_max_) also found using the *A* − *C*_c_ curve, where RuBP‐ regeneration is limiting *A*, was associated with lower diffusion resistance through mesophyll (e.g., higher *θ*_IAS_) and higher *g*_m_, and higher enzymatic activity during CO_2_ fixation and carbohydrate formation (i.e., Calvin cycle) for low latitude *J. regia* accessions. *A*_max_ was strongly associated to *V*_cmax_ and *J*_max_ (Figure S6), supporting greater biochemical and diffusional capacity for low latitude accessions which exhibit thicker leaves with greater *θ*_IAS_ and *g*_m_, and more nitrogen accumulation within leaves.

On the other hand, the photosynthetic performance seems to be unrelated to the phylogenetic history. Accessions with greater *A*_n_ and *A*_max_, such as A3 and A9, share a close evolutionary background with low‐performance accessions, like A4 and A11, but not with each other (Figure 1B). Therefore, unlike studies that reported strong phylogenetic support for water stress resistant traits like xylem cavitation vulnerability in stem and root (Willson et al., 2008) or vein development, patterning and hydraulic conductance (Brodribb et al., 2007), our results suggest that geographical variability is more strongly linked with differences in photosynthetic rate in *Juglans* accessions. Therefore, high intraspecific variability in leaf anatomical characteristics and high variation in photosynthetic performance between *J. regia* accessions are in line with their establishment across a wide range of habitats with contrasting environmental conditions. This is consistent with results of He et al. (2018b), who showed that species with lower variability in traits like specific leaf area have narrower habitat range, contrasting other studies with strong influence of phylogenetic history on traits variation (Homeier et al., 2021).

Leaf nitrogen content per unit area (Leaf N) was negatively related to latitude and supported higher *A*_n_ with greater biochemical activity. Increases in *A*_n_ related to changes in Leaf N and chlorophyll content have been reported for *Populus balsamifera* and *P. angustifolia* populations as an adaptive response to growing season length (Soolanayakanahally et al., 2009; Kaluthota et al., 2015). Latitudinal variation in photosynthetic variables have been reported more broadly across various species, however, the patterns were opposite in *Populus* spp. For example, latitudinal increases in *A*_n_ and for *P. trichocarpa* genotypes was accompanied by greater *g*_m_ through higher carbonic anhydrase activity and aquaporins functioning that were attributed to growth under shorter growing season in northern habitats (Gornall & Guy, 2007; McKown et al., 2014a, 2014b; Momayyezi & Guy, 2017, 2018).

### 4.3 Dehydration‐induced responses

As expected, dehydration negatively impacted photosynthesis in all accessions, but some exhibited greater reductions in *A*_n_ with decreases in *g*_m_, *g*_s_ and Ψ_leaflet_ (*p* = 0.007). Accessions A3, A5 and A9 exhibited similar absolute values to each other (Table S2) and had the highest photosynthetic capacity than other accessions, with the highest % reductions due to drought. A reduction in photosynthesis under dehydration was associated with decreases in PSII efficiency, which could be due to increases in photorespiration associated with increased resistance to CO_2_ diffusion through stomata and mesophyll (Busch 2020; Neto et al., 2017; Sharkey 1988). Even under dehydration, accessions A3 and A5 from lower latitudes maintained *A*_n_, *g*_m_, *θ*_IAS_, *g*_IAS_ and Leaf N higher than other accessions (Table 2), suggesting that these accessions hold potential for commercial production without increasing susceptibility to stress in absolute terms.

Dehydration reduced leaf thickness and increased *θ*_IAS_ and *g*_IAS_ by shrinking mesophyll cell size more than IAS (Table 1 and S2); this is consistent with our earlier observations for *J. regia* cv. Chandler and *J. microcarpa* (Momayyezi et al., 2022). In the current study, *g*_IAS_ increased by 13‐35% in different *J. regia* accessions under dehydration. However, its contribution to *g*_m_ (calculated as described by Niinemets & Reichstein, 2003) was 7–23% under well‐watered but decreased to 3–8% under drought across accessions, which is within the expected limiting range (3–37%) for woody perennial species with hypostomatous leaves (Harwood et al., 2021; Niinemets & Reichstein, 2003; Parkhurst & Mott, 1990; Tomás et al., 2013; Tosens et al., 2012). Additionally, reductions in the *g*_IAS_ contribution to *g*_m_ was closely and positively related to *A*_n_ (*R* ^2^ = 0.63; *p* =;0.003) and *A*_max_ (*R* ^2^ = 0.37; *p* = 0.044), suggesting reduced gas phase diffusion under stress may decrease photosynthesis further by limiting CO_2_ diffusion in liquid phase through chloroplast re‐positioning and activity of carbonic anhydrases and aquaporins (Evans et al., 2009; Miyazawa et al., 2008; Momayyezi et al., 2020; Tholen et al., 2008; Tomás et al., 2013).

### 4.4 Conclusion

We found that photosynthetic capacity in *J. regia* accessions was associated with leaf anatomical and biochemical components that impact CO_2_ diffusion. Leaves with greater porosity and *g*_IAS_ contribution to *g*_m_ exhibited the highest photosynthetic capacity at ambient and saturating CO_2_. Improved photosynthesis was supported by increased carboxylation capacity and leaf nitrogen accumulation. Higher photosynthesis across accessions was associated with frost‐free days and precipitation and temperature seasonality patterns in low‐latitude native habitats. Although *J. regia* has a limited resilience under dehydration, two of the low‐latitude accessions (e.g. A3 and A5) with the highest inherent photosynthetic capacity, sustained performance under stress. These accessions hold promise for high productivity and use in breeding programs for commercial walnut production (Figure 2).

## CONFLICT OF INTEREST

The authors declare no conflict of interest.

## Supporting information

Supplementary information.
