# Supplementary material for: Structural and functional leaf diversity lead to variability in photosynthetic capacity across a range of Juglans regia genotypes
Source: Plant Cell Environ. 2022 Jun 20;45(8):2351–65. doi: 10.1111/pce.14370 (PMC9543909; doi:10.1111/pce.14370)
Supplement: Supplementary file 1 — Supplementary information. [file PCE-45-2351-s001.docx]

**Supporting Information**

**Table S1.** Geographic data for origins of 11 *J. regia* accessions collected at the US National Germplasm Repository located at the Wolfskill Experimental Orchard.

| Accession # | Latitude (̊ N) | Longitude (E) | Elevation (m) | Plant Name | Accession name in Wolfskill repository | Location in Wolfskill | Precipitation seasonality  CV | Temperature seasonality  CV | Frost-free days |
| --- | --- | --- | --- | --- | --- | --- | --- | --- | --- |
| 1 | 31.95 | 77.10 | 1199 | Lang Thacha | DJUG0486. | C 11 14 | 84.13 | 1.98 | 365 |
| 2 | 33.71 | 73.08 | 562 | Ahkrot | DJUG0275.1 | C 3 28 | 69.25 | 2.42 | 365 |
| 3 | 35.23 | 75.96 | 2637 | 880638 | DJUG0274.4 | C 3 27 | 49.42 | 2.98 | 295 |
| 4 | 35.60 | 72.65 | 4089 | 880432 | DJUG0260.4 | C 2 14 | 40.04 | 2.78 | 280 |
| 5 | 38.87 | - 6.97 | 170 | Badajoz | DJUG0420. | C 4 36 | 66.20 | 2.18 | 365 |
| 6 | 41.27 | 80.23 | 1131 | Aksu 81 | DJUG0377.8 | C 8 32 | 55.44 | 3.95 | 234 |
| 7 | 44.49 | 34.16 | 10 | Op Sdlg/paperovii | DJUG0188.1 | A 2 34 | 21.96 | 2.90 | 286 |
| 8 | 49.83 | 35.61 | 121 | DJUG 566 | DJUG0566.4 | C 16 8 | 29.74 | 3.56 | 253 |
| 9 | 50.01 | 22.22 | 238 | Nn 88 Godyn | DJUG0413. | C 4 28 | 39.33 | 2.75 | 258 |
| 10 | 50.75 | 33.50 | 111 | DJUG 606 | DJUG0606 | C 20 2 | 21.93 | 3.48 | 253 |
| 11 | 52.22 | 21.01 | 114 | R 8/6 | DJUG0411. | C 13 38 | 31.64 | 3.27 | 269 |

Temperature (°C)

Precipitation (mm)


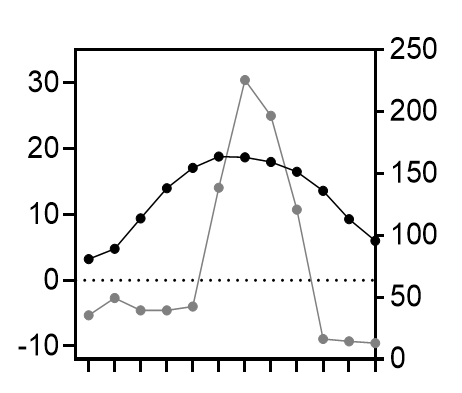

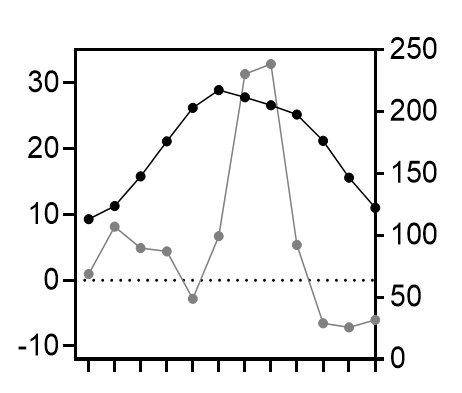

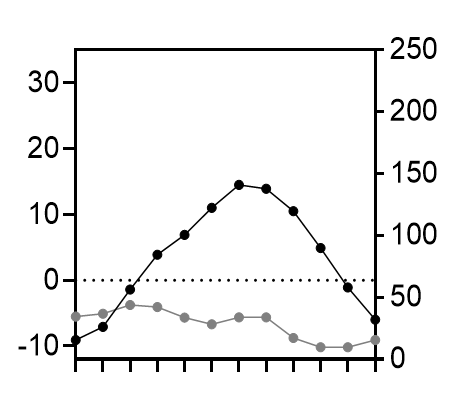

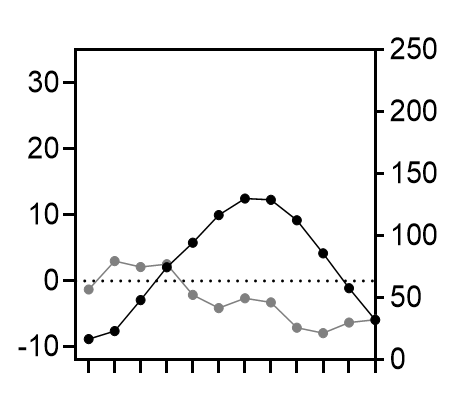

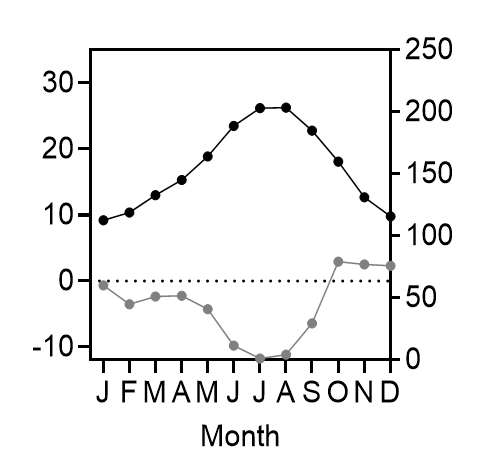

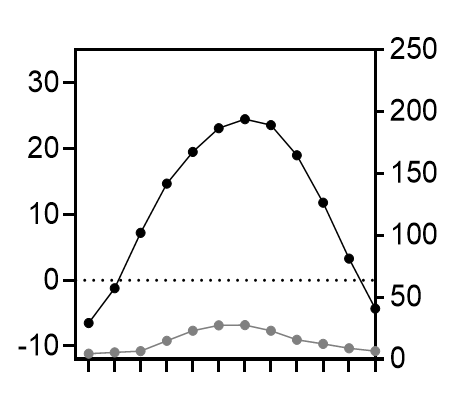

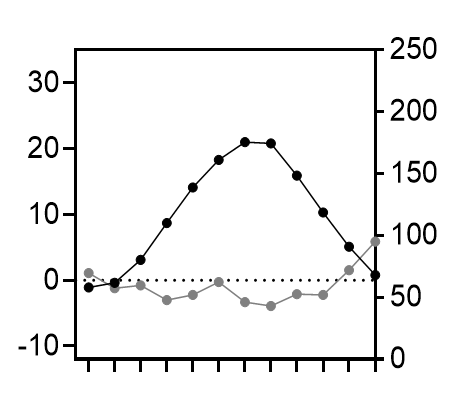

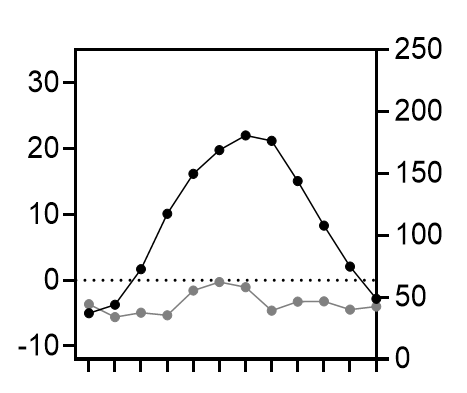

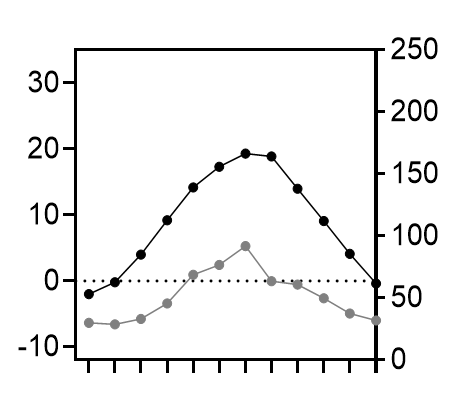

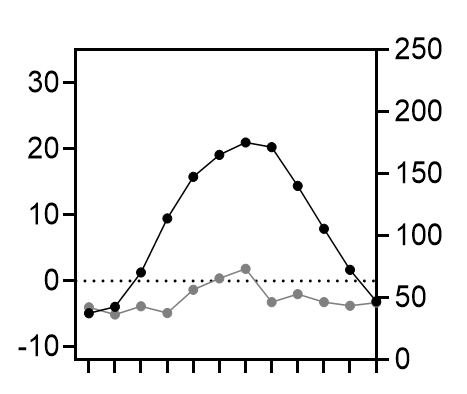

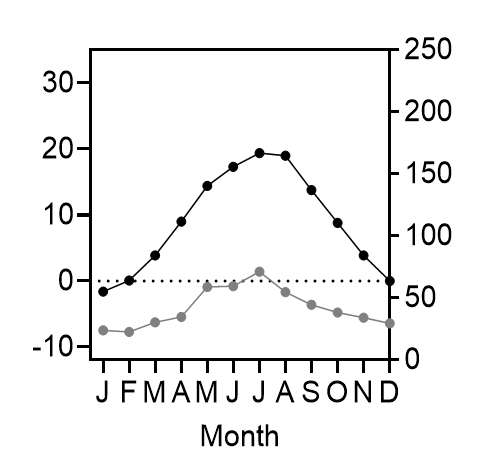


A1

A2

A3

A4

A5

A6

A7

A8

A9

A10

A11

**Figure S1.** Walter-Lieth climate diagrams for 11 *J. regia* habitats. The diagrams present average monthly temperature (black) and annual precipitation (gray). Sources: <https://climatecharts.net/>, <https://climateknowledgeportal.worldbank.org/download-data>. Latitude is positively correlated with the temperature seasonality (*P* = 0.049), and negatively related to precipitation seasonality (*P* = 0.004), and frost-free days (*P* = 0.016). As expected, low-latitude habitats showed more frost-free days and higher variability in precipitation seasonality (Zhong *et al.* 2017; Marelle *et al.* 2018; Liu *et al.* 2018).

Figure S2. Light response curves, relationship between net assimilation rate (*A*_n_, μmol CO_2_ m^-2^ s^-1^) and PPFD at 35, 50, 200, 400, 800, 1000, 1200, 1500 μmol m^-2^ m^-1^ levels at CO_2_ of 400 µmol mol^-1^ for 11 *J. regia* accessions (n =1).

**Figure S3.** Correlation between mesophyll conductance (*g*_m_, mol CO_2_ m^-2^ s^-1^) obtained from chlorophyll fluorescence and *A*_n_-*C*_i_ curve methods for each of 11 *J. regia* accessions under well-watered treatment using mean values (± SE, n = 5).


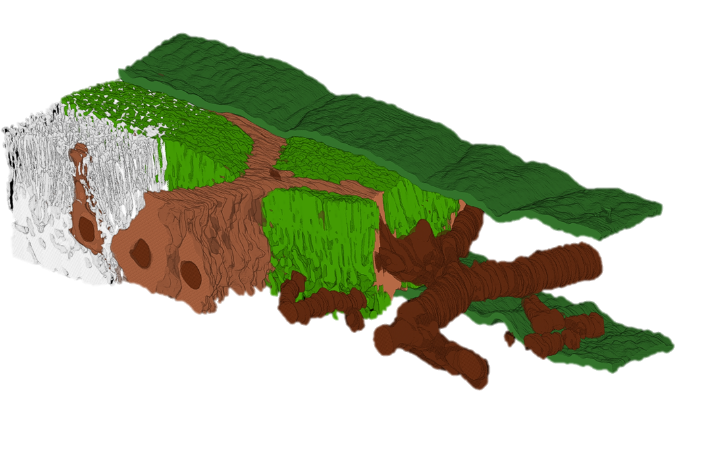

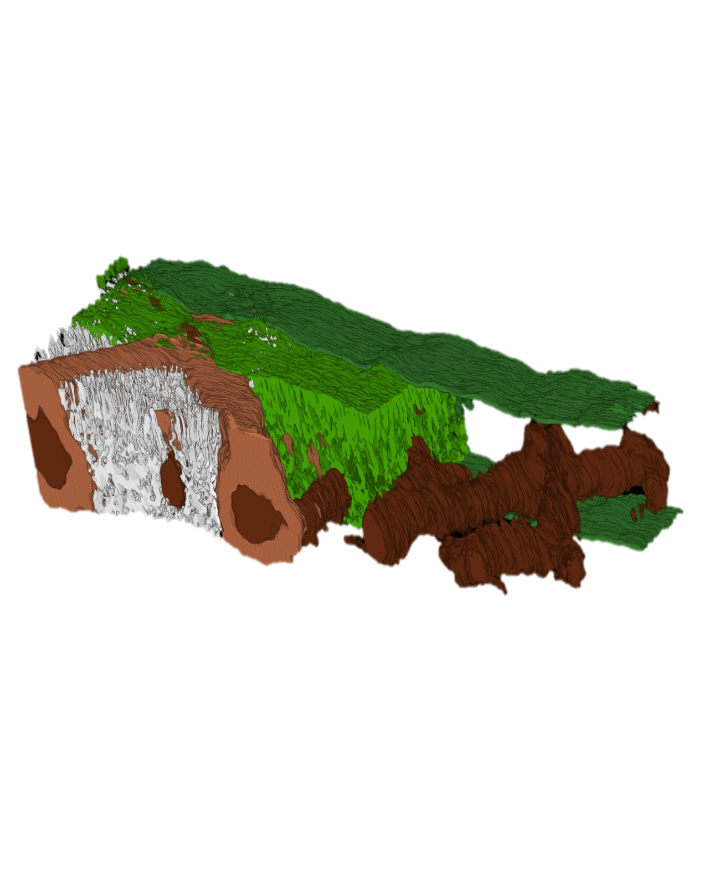


**High porosity**

**A11**

**A 1**

**Low porosity**

**Epidermis**

**Mesophyll cell**

**Bundle sheath**

**Vein**

**Airspace**

**Figure S4.** 3D projection of whole leaf for A1 and A11, high and low mesophyll porosity accessions, respectively. Each color represents a unique tissue; dark green for epidermises, light green for mesophyll cells both palisade and spongy cells, white for mesophyll airspace, light brown for bundle sheath extension, and dark brown for vein.


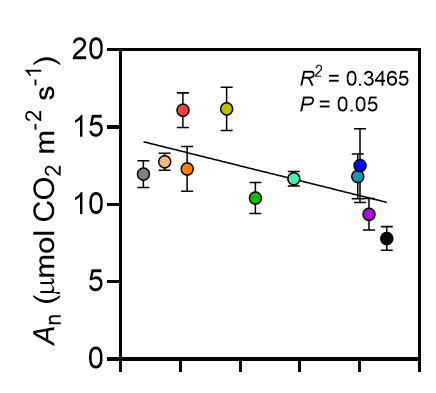

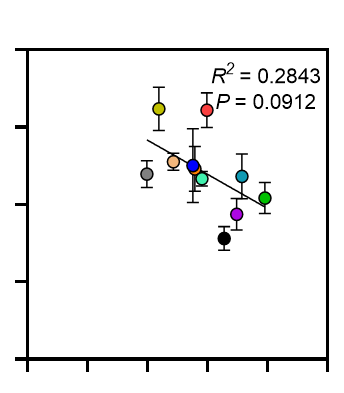

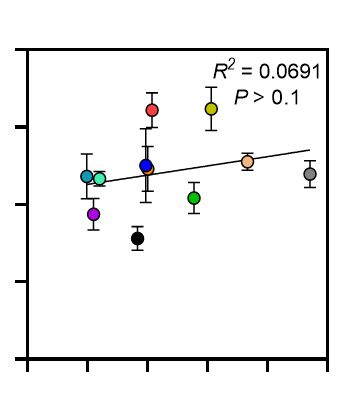

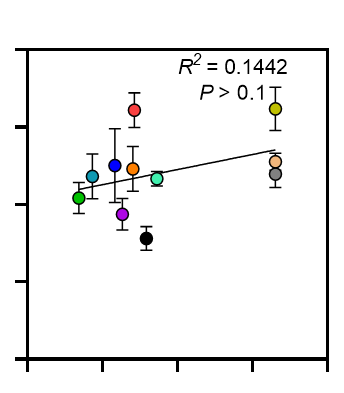

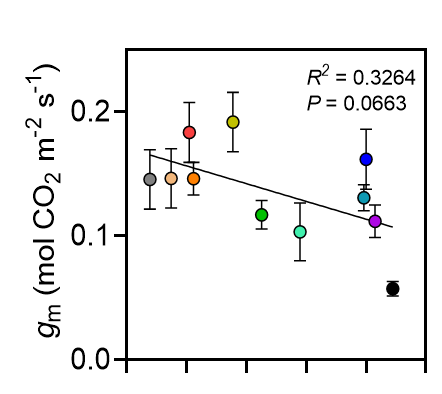

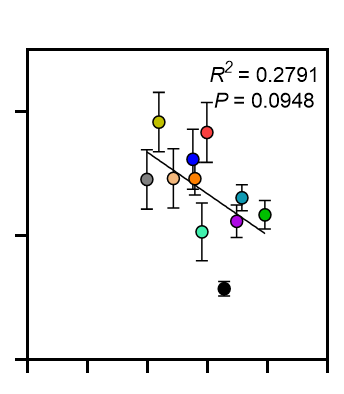

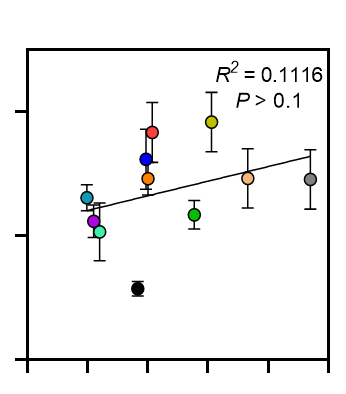

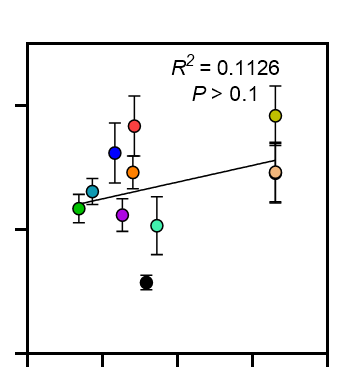

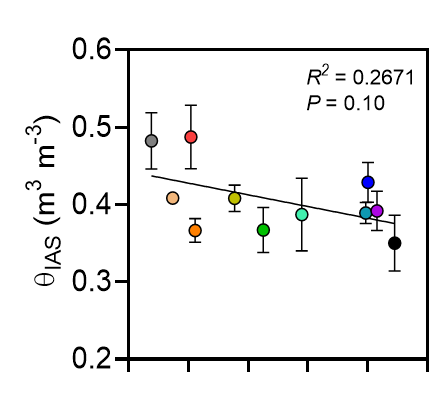

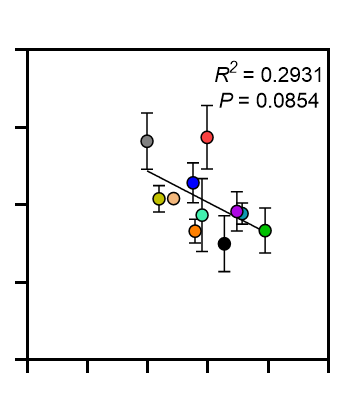

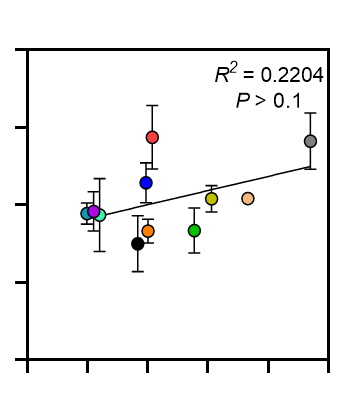

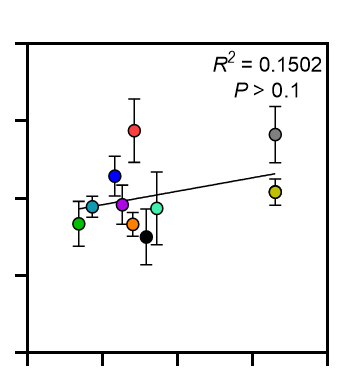

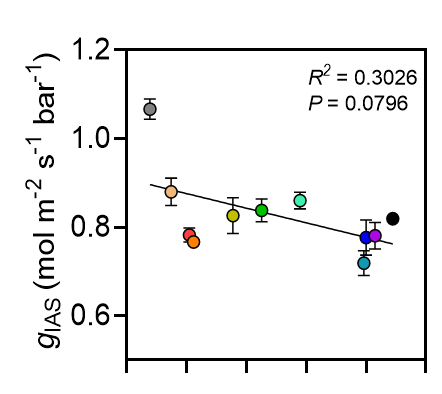

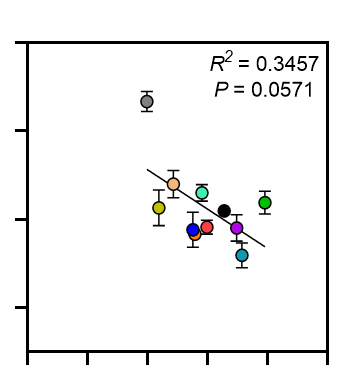

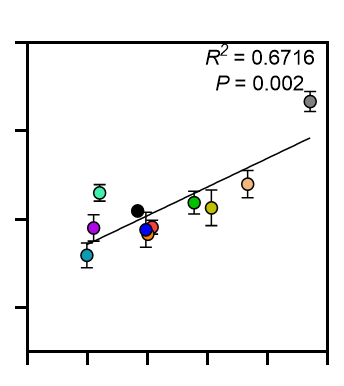

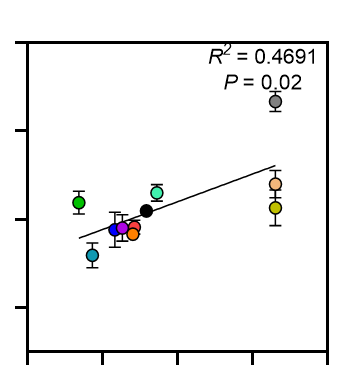

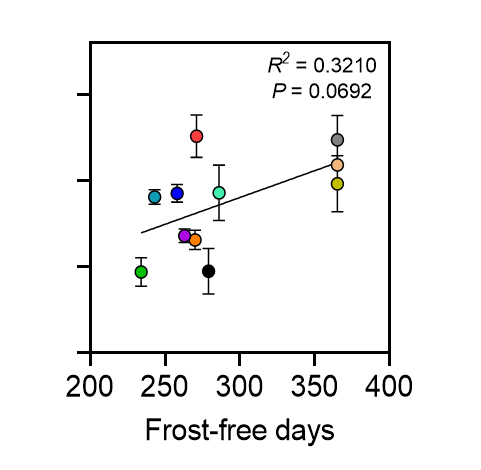

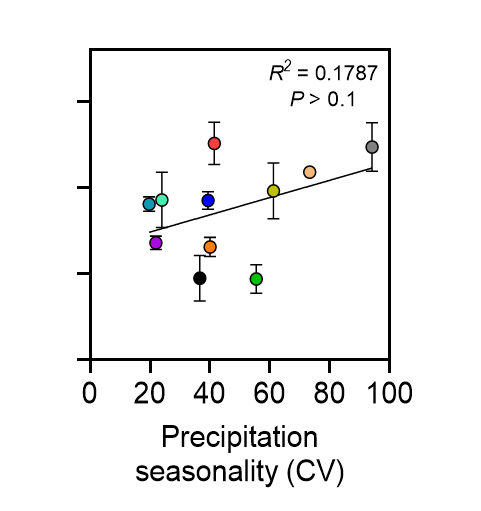

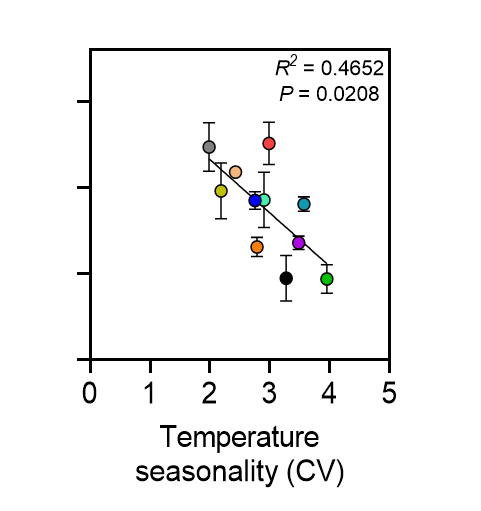

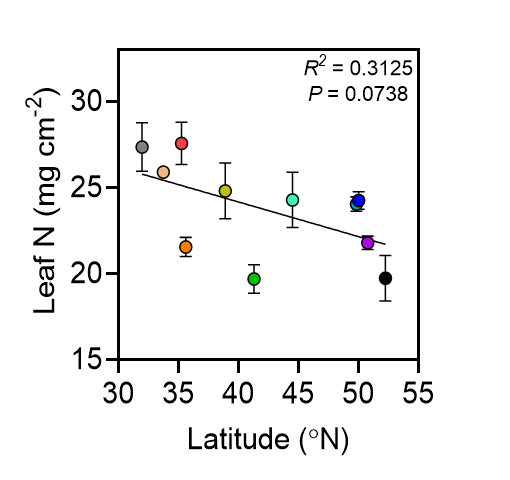

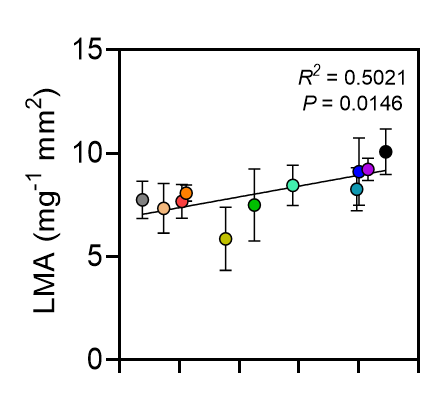

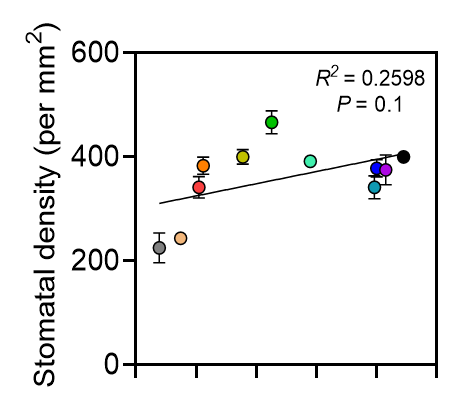

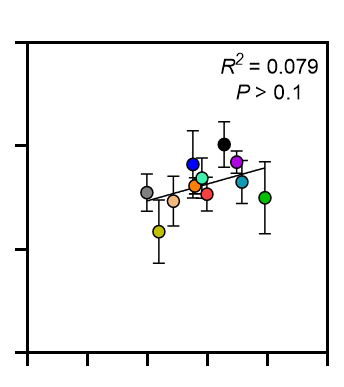

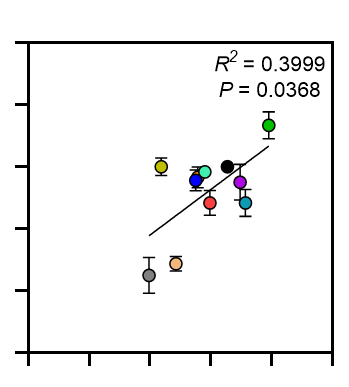

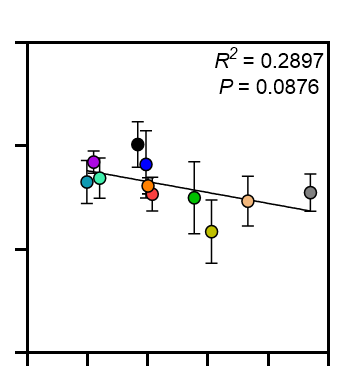

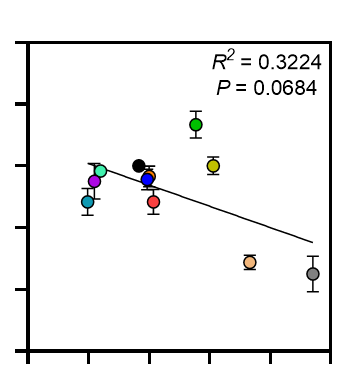

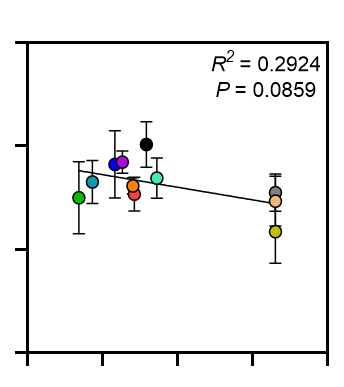

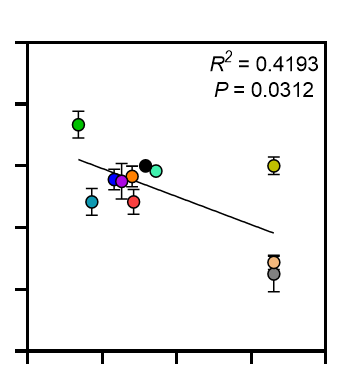


**Figure S5.** Relationship between net assimilation rate (*A*_n_, μmol CO_2_ m^-2^ s^-1^), mesophyll porosity (θ_IAS_, m^3^ m^-3^), intercellular airspace conductance (*g*_IAS_, mol CO_2_ m^-2^ s^-1^ bar ^-1^), mesophyll conductance from fluorescence method (*g*_m_, mol CO_2_ m^-2^ s^-1^), stomatal density (per mm^2^), leaf mass per unit area (LMA, mg cm^-2^), and leaf nitrogen per unit area (Leaf N, mg cm^-2^) using mean values (± SE, n = 5) and latitude, temperature seasonality, precipitation seasonality and frost-free days in habitats for 11 *J. regia* accessions.

**Figure S6.** Assimilation rate at saturating CO_2_ (*A*_max_, μmol CO_2_ m^-2^ s^-1^) relationship with mesophyll porosity (θ_IAS_, m^3^ m^-3^), leaf thickness (*L*_leaf_, μm), and leaf nitrogen per unit area (Leaf N, mg cm^-2^), maximum carboxylation rate (*V*_cmax_) and maximum electron transport rate (*J*_max_) in 11 *J. regia* accessions using mean values (± SE, n = 5).


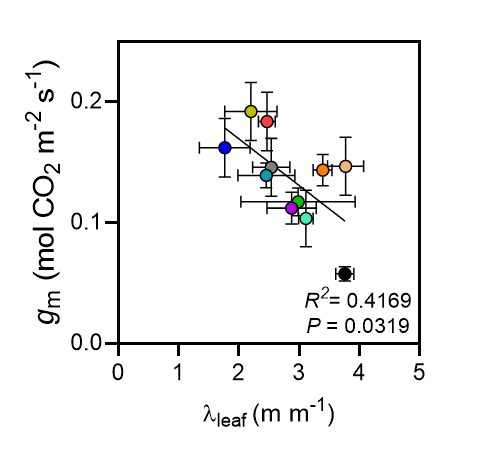

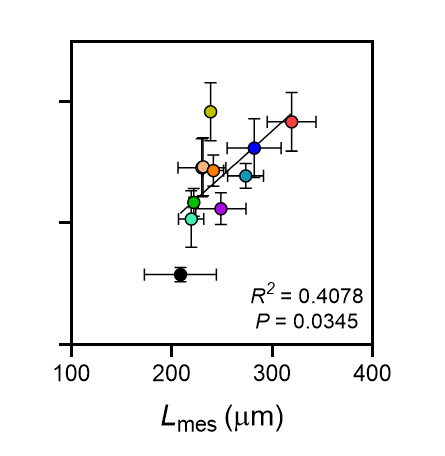

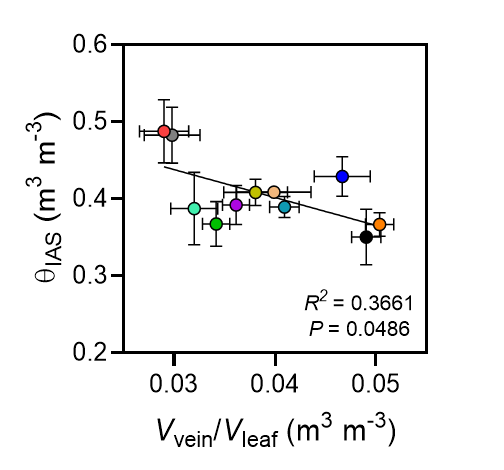

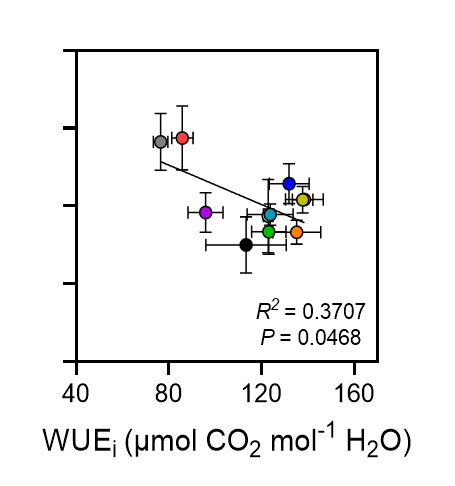


**Figure S7.** Mesophyll conductance from fluorescence method (*g*_m_, mol CO_2_ m^-2^ s^-1^) relationship with lateral path lengthening (λ_leaf_, m m^-1^), mesophyll thickness (*L*_mes_, μm), and relationship between mesophyll porosity (θ_IAS_, m^3^ m^-3^), vein volume to leaf volume ratio (*V*_vein_/*V*_leaf_, m^3^ m^-3^) and intrinsic water use efficiency (WUE_i_, μmol CO_2_ mol^-1^ H_2_O) in 11 *J. regia* accessions under well-watered condition using mean values (± SE, n = 5).

**Table S2.** Absolute values for physiological and anatomical variables under dehydration treatment. Different accessions numbers are used to show significant differences from each other using mean values (± SE) over five replications at *P* < 0.002.

| Accession # | *A*_n_ | *g*_s_ | *g*_m_ | Ψ_leaflet_ | *L*_leaf_ | θ_IAS_ | *g*_IAS_ |
| --- | --- | --- | --- | --- | --- | --- | --- |
| A 1  | 9.49 ± 0.69  (A9,10,11) | 0.11 ± 0.04 (A11) | 0.09 ± 0.01  (A5,11) | -1.05 ± 0.05 (A2,4,5,9,10,11) | 224.50 ± 6.93  (A3) | 0.53 ± 0.08  ns | 1.28 ± 0.23  ns |
| A 2  | 7.96 ± 1.02 | 0.04 ± 0.01  ns | 0.07 ± 0.02  ns | -1.40 ± 0.03  (A1,6) | 223.35 ± 7.59  (A3) | 0.49 ± 0.06  ns | 1.32 ± 0.26  ns |
| A 3  | 7.92 ± 1.16  ns | 0.06 ± 0.02  ns | 0.08 ± 0.03  ns | -1.14 ± 0.13 (A5,9,10) | 308.75 ± 15.28  (A1,2,4,5,6,7,10,11) | 0.62 ± 0.08  ns | 1.21 ± 0.12  (A11) |
| A 4  | 7.63 ± 0.78  ns | 0.05 ± 0.01  ns | 0.06 ± 0.01  ns | -1.42 ± 0.04 (A1,6,8) | 240.68 ± 10.27  (A3) | 0.43 ± 0.01 ns | 0.98 ± 0.15  ns |
| A 5  | 7.00 ± 1.40  ns | 0.04 ± 0.01  ns | 0.04 ± 0.01  (A1) | -1.63 ± 0.04  (A1,3,6,7,8) | 238.29 ± 12.68  (A3) | 0.46 ± 0.01  ns | 1.10 ± 0.02  ns |
| A 6  | 6.64 ± 0.61  ns | 0.05 ± 0.01  ns | 0.05 ± 0.01  ns | -0.97 ± 0.04  (A2,4,5,9) | 207.72 ± 4.66 (A3,8,9) | 0.42 ± 0.03  ns | 1.22 ± 0.26  ns |
| A 7  | 9.35 ± 1.67  ns | 0.07 ± 0.02  ns | 0.08 ± 0.03  ns | -1.31 ± 0.02  (A5,7,9,10,11) | 228.67 ± 12.57  (A3) | 0.43 ± 0.02  ns | 0.99 ± 0.05 ns |
| A 8  | 7.01 ± 0.94  ns | 0.08 ± 0.02  ns | 0.06 ± 0.01  ns | -1.11 ± 0.06  (A4,5,9,10,11) | 267.81 ± 8.84  (A11) | 0.41 ± 0.01 ns | 0.91 ± 0.19  ns |
| A 9  | 5.98 ± 0.84  (A1) | 0.03 ± 0.01  ns | 0.06 ± 0.01  ns | -1.50 ± 0.09  (A1,3,6,8) | 274.79 ± 6.87  (A11) | 0.51 ± 0.04  ns | 1.09 ± 0.12 ns |
| A 10  | 5.16 ± 1.34  (A1) | 0.04 ± 0.01  ns | 0.06 ± 0.01  ns | -1.48 ± 0.03  (A1,3,8) | 253.49 ± 15.09  (A3) | 0.42 ± 0.01  ns | 0.89 ± 0.09  ns |
| A 11  | 5.03 ± 0.81  (A1) | 0.02 ± 0.01  (A1) | 0.03 ± 0.01  (A1) | -1.42 ± 0.06  (A1,8) | 208.50 ± 10.79  (A3) | 0.39 ± 0.02  ns | 1.04 ± 0.24  (A3) |
| *P* value | < 0.002 | < 0.002 | < 0.002 | < 0.002 | < 0.002 | < 0.002 | < 0.002 |

*A*_n_, net assimilation rate (µmol CO_2_ m^-2^ s^-1^); *g*_s_, stomatal conductance (mol m^-2^ s^-1^); *g*_m_, mesophyll conductance obtained from chlorophyll fluorescence method (mol CO_2_ m^-2^ s^-1^); Ψ_leaflet_, leaflet water potential (MPa); *L*_leaf_, leaf thickness (μm); θ_IAS_, mesophyll porosity (m^3^ m^-3^), *g*_IAS_, intercellular airspace conductance (mol m^-2^ s^-1^ bar^-1^).

**References**

**Liu Q, Piao S, Janssens IA, Fu Y, Peng S, Lian X, Ciais P, Myneni RB, Peñuelas, Wang T. 2018.**Extension of the growing season increases vegetation exposure to frost. *Nature Communications* **9:**426. https://doi.org/10.1038/s41467-017-02690-y.

**Marelle L, Myhre G, Hodnebrog Ø, Sillmann J, Samset BH. 2018.** The changing seasonality of extreme daily precipitation. *Geophysical Research Letters* **45:** 11352-11360.

**Zhong S, Yu L, Winkler JA, Tang Y, Heilman WA, Bian X. 2017.** The impact of climate change on the characteristics of the frost-free season over the contiguous USA as projected by the NARCCAP model ensembles. *Climate Research* **72:** 53-72.
